# Dirichlet process mixture models to impute missing predictor data in counterfactual prediction models: an application to predict optimal type 2 diabetes therapy

**DOI:** 10.1186/s12911-023-02400-3

**Published:** 2024-01-08

**Authors:** Pedro Cardoso, John M. Dennis, Jack Bowden, Beverley M. Shields, Trevelyan J. McKinley, the MASTERMIND Consortium

**Affiliations:** University of Exeter, Medical School, Exeter, England

**Keywords:** Dirichlet process mixture model, Treatment selection model, Precision medicine, Type 2 diabetes, Bayesian modelling

## Abstract

**Background:**

The handling of missing data is a challenge for inference and regression modelling. A particular challenge is dealing with missing predictor information, particularly when trying to build and make predictions from models for use in clinical practice.

**Methods:**

We utilise a flexible Bayesian approach for handling missing predictor information in regression models. This provides practitioners with full posterior predictive distributions for both the missing predictor information (conditional on the observed predictors) and the outcome-of-interest. We apply this approach to a previously proposed counterfactual treatment selection model for type 2 diabetes second-line therapies. Our approach combines a regression model and a Dirichlet process mixture model (DPMM), where the former defines the treatment selection model, and the latter provides a flexible way to model the joint distribution of the predictors.

**Results:**

We show that DPMMs can model complex relationships between predictor variables and can provide powerful means of fitting models to incomplete data (under missing-completely-at-random and missing-at-random assumptions). This framework ensures that the posterior distribution for the parameters and the conditional average treatment effect estimates automatically reflect the additional uncertainties associated with missing data due to the hierarchical model structure. We also demonstrate that in the presence of multiple missing predictors, the DPMM model can be used to explore which variable(s), if collected, could provide the most additional information about the likely outcome.

**Conclusions:**

When developing clinical prediction models, DPMMs offer a flexible way to model complex covariate structures and handle missing predictor information. DPMM-based counterfactual prediction models can also provide additional information to support clinical decision-making, including allowing predictions with appropriate uncertainty to be made for individuals with incomplete predictor data.

**Supplementary Information:**

The online version contains supplementary material available at 10.1186/s12911-023-02400-3.

## Background

Prediction models are being increasingly deployed to support clinical decision making in healthcare. These models typically take a set of predictor (explanatory) variables—for example, a set of routinely collected clinical features—and then use these to directly predict a key outcome-of-interest. Counterfactual prediction models provide a recent extension to standard prediction models by supporting estimation of differential effects of treatments conditional on predictor variables. Models predicting such differential treatment effects have the potential to inform precision medicine approaches aiming to target specific treatment(s) to individual patient most likely to benefit based on their characteristics. Such models are often termed ‘treatment selection models’. A major challenge when building and implementing both types of clinical prediction models in practice is dealing with missing predictor data. This is particularly pertinent if a given model includes predictor variables that are informative about the outcome, but are not routinely collected, such as diagnostic test information. In this case it is not possible to produce predictions unless the model has a way to impute the missing information, thus severely hampering the utility of such models when deployed in clinical practice.

In the context of counterfactual prediction models, dealing robustly with missing data is one of the key priority research areas highlighted in the recent Predictive Approaches to Treatment effect Heterogeneity (PATH) statement, a document outlining principles, criteria and key considerations for predictive approaches of heterogeneity of treatment effects [1]. In particular, this manuscript aims to contribute to the requirement to: “Determine methods to permit models predicting treatment effect to cope with missing data in clinical practice” [1]. In this study, we extend an exemplar recently published counterfactual treatment selection model for type 2 diabetes (T2D) that predicts optimal treatment for individual patients based on routinely collected clinical features [2]. We build on this previous work (which was based on a complete-case analysis) to explore the potential for using cutting-edge Bayesian approaches to handle missing data during both model development and model deployment.

There are three types of missing data that occur during data collection: data can be missing completely-at-random (MCAR) when the missing values can be thought of as a random sub-sample of the actual values; missing-at-random (MAR) when the pattern of missingness is predictable from other variables in the dataset; and missing-not-at-random (MNAR) when the missingness is non-random, and it is not predictable from other variables in the dataset [3]. Due to difficulties modelling missing data, many prediction models use only complete-case information, which can lead to biases unless the data are MCAR [4]. The complete-case analysis also throws away potentially informative data from individuals with incomplete records. Since clinical data is often incomplete, then without a way to handle missing predictors, it would also not be possible to predict for any new patient with incomplete predictor information unless there was a way to robustly impute the missing data.

A common approach for dealing with missing predictors, under the assumption of MAR, involves the use of multiple imputation (MI), commonly through chained equations (MICE) [3, 5]. Whilst powerful and straightforward to implement, MICE has some limitations. Unless the chained equations have a carefully chosen hierarchical dependence structure, these do not always provide a probabilistically consistent joint probability model for the predictors [6]. Another limitation of multiple imputation involves the propagation of uncertainties to the parameters and predictions, since this only occurs through the standard errors and therefore does not provide the richness of information afforded by full Bayesian posterior (predictive) distributions.

Furthermore there are various challenges with the deployment of prediction models using MI in clinical practice [7]. For example, it is often recommended that the response variable should be included in the MI model to improve the accuracy of the fitted model [8]; however, by definition the response variable will not be observed if using the model to predict the outcome for a new individual. As such, to produce predictions for new individuals with missing data, then MI would have to be conducted without using the response variable, thus potentially reducing the accuracy of the predictions [8]. For more discussion of these challenges, please see [7].

Bayesian approaches offer principled ways to deal with the issues described above. The implementation of a hierarchical Bayesian prediction model, in which the response model is dependent on a flexible joint probability model for the predictor variables, facilitates both inference and prediction in the presence of missing data [9]. Firstly, this defines a joint model for the response and predictor distributions, meaning that information on the response variable is naturally incorporated into posterior estimates of missing predictors during model fitting. Furthermore, one can estimate full posterior predictive distributions for new individuals empirically, using posterior samples obtained via e.g MCMC methods. This has a useful side-effect in that for a given set of posterior samples, it is possible to generate samples from a corresponding predictive distribution for a new individual without requiring access to the original dataset. This is in contrast to models affording an analytical solution, such as classical linear regression, where confidence and prediction intervals are typically functions of the original data. This makes general deployment of such models easier in the case where the original training data is under confidentiality restrictions with restricted access.

To this end, Dirichlet process mixture models (DPMMs) [10] provide a powerful way to model the joint (complex) distribution of a set of predictor variables. They do this by modelling complex non-standard distributions through a mixture of simpler, more tractable distributions (see Table 1). This approach can readily capture non-standard features such as non-linearities and heteroscedasticity across different dimensions. Furthermore, it can handle mixtures of both numerical and categorical predictors [11–15]. This allows for fully probabilistic inference under the MCAR and MAR assumptions, contributing towards addressing some of the points raised in the PATH statement highlighted above.

**Table 1:** Dirichlet process mixture models (DPMMs)

A DPMM is commonly used as a prior distribution over the components of a (possibly multivariate) mixture model of unknown complexity [16]. A DPMM consists of a theoretically infinite number of components, where each component is parameterised by a specific functional form with component-specific parameters. Instead of fitting an infinite number of components, we use a truncated DPMM with a maximum number of components *K*, based on the assumption the optimal number of components is lower than the set limit [17]. In turn, the computational demands for fitting the model are reduced. The value of *K* should increase as the complexity of the data increases, but its suitability can be checked post model fit.	
The DPMM thus defines a weighted sum of *K* component densities [18]. The component densities are restricted to particular parametric classes of densities that are assumed to be appropriate for the data at hand. We define $f_k\left( \textbf{X} \mid \varvec{\Theta }_k \right)$ as the $k^{\text{ th }}$ component density, with $\varvec{\Theta }_k$ representing the component parameters. A *K* component mixture density is defined as:	
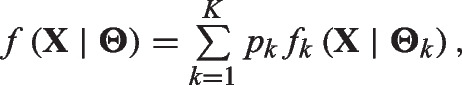	
where $p_k$ are component-specific weights such that $\sum \nolimits _{k = 1}^K p_k = 1$ [19].	
For $J_{C}$ continuous predictors, we use mixtures of multivariate Gaussian distributions with $J_C$ dimensions, the cluster-specific parameters for component *k* ($k = 1,\dots ,K$) are given by $\left( \varvec{\mu } _{k}, {\varvec{\Sigma }} _{k}\right)$, where $\varvec{\mu }_{k}$ is a $J_C$-vector of means and $\varvec{\Sigma }_{k}$ is a $(J_C \times J_C)$ covariance matrix. For $J_D$ categorical predictors, we use mixtures of categorical probability mass functions, where the number of categories for a covariate *j* ($j = 1,\dots ,J_D$) is $K_{j}$, the component-specific parameters are the probabilities of belonging to each category, given by $\varvec{\phi }_{k} = (\varvec{\phi }_{k1}, \varvec{\phi }_{k2}, ..., \varvec{\phi }_{kJ_D})$ with $\varvec{\phi }_{kj}=(\phi _{kj1},\phi _{kj2},\dots ,\phi _{kjK_{j}})$ and $\sum \nolimits _{l = 1}^{K_j} \phi _{kjl} = 1$. The model in this paper is given as a mixture of continuous and categorical variables, since $\textbf{X}_{i} = \left( \textbf{X}^{C}_{i},\textbf{X}^{D}_{i}, X^T_i\right)$, with $\textbf{X}^{C}_{i}$ representing the continuous predictors, $\textbf{X}^{D}_{i}$ corresponding to the categorical predictors and $\textbf{X}^{T}_{i}$ representing the treatment taken. Hence in the notation of the Study overview section, $\varvec{\Theta } = \left( \varvec{\Theta }_1, \dots , \varvec{\Theta }_K, p_1, \dots , p_K\right)$, where the component-specific parameters are given by $\varvec{\Theta }_{k} = (\varvec{\mu }_{k}, {\varvec{\Sigma }}_{k}, \varvec{\phi }_{k})$.	
A latent variable, $Z_{i} = 1, \dots , K$, is used to assign individual data points to different components of the mixture model, and we assume independence between continuous and categorical components conditional on the cluster allocations [11, 12]. Thus the probability density for individual *i*, given $Z_i$ is:	
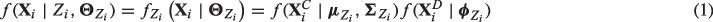	
More details on the component densities, prior distributions, and how to sample from the DPMM are given in the Supplementary Materials.	

For an extensive exploration of Bayesian non-parametric methods and missing data, see [20]. DPMMs have also been established as a clustering mechanism [21], used in multiple imputation [14, 15, 22–26] and used as a joint model in a specific type of hierarchical regression model known as profile regression [11, 12]. In *profile regression*, a DPMM is fitted to a set of predictor variables providing a probabilistic mapping of each individual observation to different component clusters within the DPMM. The cluster labels are then used as predictor variables in a hierarchical regression model targeting the response. Hence, profile regression ultimately captures the relationship between covariate profiles (as modelled through the cluster structure of the DPMM) and the response variable-of-interest [11]. In contrast, in this manuscript, we use a DPMM to explicitly model the joint distribution of the predictors (given the observed data), which can then impute missing data directly and feed these into the outcome model-of-interest. This is similar to the approach used in [27], who develop a flexible hierarchical framework that can be applied to treatment selection models that uses an enriched Dirichlet process [28, 29] as a means of capturing the complex joint distribution of the predictors *and* the response, providing a means of imputing missing data under a MCAR/MAR assumption. These methods are highly flexible, and can generate estimates of the response regression function indirectly from the joint predictive density (an idea originating in [30]; see e.g. [29] and references therein for a more detailed discussion). However, they are technically challenging to implement in practice without bespoke code.

The original motivation for the work in this paper was to augment currently developed and validated models to enable prediction for new patients with incomplete predictor data, and thus facilitate their deployment in routine clinical practice. Furthermore we wanted to be able to develop these models in general-purpose Bayesian analysis software, to make these ideas more accessible to a wider audience and to make them applicable to a wide-class of pre-existing models. As such we decided to use a simpler approach where we use the DPMM to model the joint distribution of the predictor variables as a means of imputing missing data, and then use the aforementioned spline-based treatment selection model in T2D [2] as our outcome model-of-interest, which combines routine clinical features of each patient to predict future blood glucose (HbA1c) levels under two commonly prescribed treatments, SGLT2i and DPP4i. We will henceforth refer to this model as the SGLT2i-DPP4i treatment selection model. The analysis identified marked heterogeneity in HbA1c outcome that is predicted by baseline patient characteristics: age-at-treatment, baseline HbA1c, body mass index (BMI), estimated glomerular filtration rate (eGFR), alanine aminotransferase (ALT) measurements and previous treatment history. These predictions can determine which treatment is likely to be optimal for an individual patient based on their characteristics. We carefully validated the model in hold-out data, and well as the development data.

Furthermore, we show how the model can also be used to identify how predictions of the response or treatment selection outcomes might change if any of the missing predictor variable(s) were available, and if so, which ones (if any) would be the most useful to collect. We note that this latter feature could be particularly useful for modelling non-routinely collected diagnostic test information, such that one could examine how predictions might change if the test were to be conducted, and hence could be used to inform whether it is worthwhile to perform the test or not. We also provide code that enables this approach to be implemented in the general-purpose Bayesian modelling package NIMBLE, within the R statistical language (which by default does not currently allow for the conditional sampling of missing dimensions of multivariate nodes, which is required for this approach to work).

## Methods

### Study overview

The focus here is on enhancing the previously described SGLT2i–DPP4i penalised linear regression model designed to predict likely glucose-lowering response (HbA1c) in people with T2D initiating SGLT2i or DPP4i therapy [2]. For an individual patient, the difference in the predicted outcome for each treatment is then used to infer which is likely to be optimal. Such models are labelled treatment selection models [31].

The model uses interaction effects between the treatment and the other predictors to allow for differential responses to treatment given a set of predictors. For a new individual with a specific set of characteristics, mean predictions of the HbA1c change at 6/12 months post-treatment can be made under both treatments and then the difference can be derived. We note that there is considerable residual uncertainty in the fitted models, and as such [2, 31] only use point predictions when making comparisons. With this in mind, predictions should be interpreted as the expected difference for an individual with a specific set of predictors under the two treatments, commonly named the conditional average treatment effect (CATE). In the Bayesian model below, we follow the same principle, where posterior predictive distributions are for the expected treatment effect for an individual with a given set of characteristics.

In the model description below, we use $\textbf{Y}$ to denote the outcome variable and $\textbf{X}$ to denote the set of predictor variables. Hence, $f(\textbf{Y}\mid \textbf{X},\varvec{\psi })$ corresponds to the likelihood function from the regression model of Dennis et al. [2], with parameters $\varvec{\psi }$; $f(\textbf{X}\mid \varvec{\Theta })$ corresponds to the joint probability density function for the DPMM, given parameters $\varvec{\Theta }$ (see Table 1); and $f(\varvec{\psi },\varvec{\Theta })$ corresponds to the prior distribution for the parameters, then our Bayesian hierarchical model has posterior distribution:

2 $$\begin{aligned} f(\varvec{\psi },\varvec{\Theta }, \textbf{X}^m\mid \textbf{X}^o,\textbf{Y})\propto f(\textbf{Y}\mid \textbf{X}^o,\textbf{X}^m,\varvec{\psi }) f(\textbf{X}^o,\textbf{X}^m\mid \varvec{\Theta }) f(\varvec{\psi },\varvec{\Theta }). \end{aligned}$$

where $\textbf{X}^o$ corresponds to all observed covariates, the full set of covariates for complete individuals and the subset of covariates that were observed for those with missingness, and $\textbf{X}^m$ represents the unobserved covariates, but not the observed values for the incomplete patients (see the Supplementary Materials Section S1 for more details). If fitting to complete-case data only, then the posterior simplifies to Equation (S.3) in the Supplementary Materials. We refer the reader to [9] for a comprehensive reference for Bayesian hierarchical models.

We fit this model using Markov chain Monte Carlo (MCMC) with the package NIMBLE [32, 33] (version 0.12.2) in the software **R** [34] (version 4.0.3). We note that in the current version of NIMBLE, using the in-built samplers, it is not possible to update individual dimensions of a multivariate node. However, NIMBLE does allow users to specify their own custom MCMC samplers, and so in order to implement model (2) we wrote our own custom samplers, and provide complete code for model fitting and prediction at https://github.com/PM-Cardoso/DPMM-tsm. We cannot share the original data due to confidentiality constraints, but we provide an example of using the code to fit a model to a smaller synthetic dataset.

### Data/Study population

The analysis in this paper is performed with anonymised routinely collected, population-representative UK primary care electronic healthcare records from Clinical Practice Research Datalink (CPRD) GOLD (July 2019 download) [35], selecting users of SGLT2i and DPP4i therapy after January 1st, 2013. We used the same dataset as previously described by Dennis et al. [2] in the initial model development study and extracted the same clinical feature predictor variables (age-at-treatment, baseline HbA1c, BMI, eGFR, ALT, the number of previously prescribed glucose-lowering therapies, the current therapy and its duration, the number of ongoing prescribed treatments) and HbA1c outcome. The dataset is split into two groups: the development group (60% of the dataset corresponding to 16,126 patients) and the validation group (40% of the dataset corresponding to 10,751 patients). Compared to the dataset used to fit the original model [2], there are an extra 2,057 patients in the development group and 1,375 patients in the validation group with incomplete predictor variables (Table 2). We fit to the development dataset and undertake both internal and external validation.

**Table 2:** Summaries of differential predictors and biomarkers used in the model for the development and validation datasets. (SD) [percentage of missing values]

	Development Dataset		Validation Dataset	
Drug Taken	DPP4-inhibitor	SGLT2-inhibitor	DPP4-inhibitor	SGLT2-inhibitor
	(n = 9,974)	(n = 6,152)	(n = 6,650)	(n = 4,101)
Age (years)	63.9 (10.8)	59.9 (9.1)	65.0 (10.7)	60.2 (9.3)
Number of Past Drugs				
2	3,884	1,167	2,556	731
3	3,653	1,693	2,457	1,115
4	972	1,569	683	1,045
5+	186	945	117	672
Number of Current Drugs				
0	523	149	309	93
1	5,078	2,191	3,424	1,418
2	3,000	2,449	1,993	1,643
3+	94	585	87	409
HbA1c (mmol/mol)	72.9 (13.5)	76.6 (14.2)	72.6 (13.2)	77.1 (14.1)
eGFR (mL/min/1.3m2)	83.1 (17.4) [0.2%]	88.8 (14.7) [0.3%]	84.9 (17.2) [0.2%]	88.6 (14.8) [0.4%]
ALT (IU/L) (logged)	3.3 (0.5) [10.0%]	3.4 (0.5) [10.4%]	3.3 (0.5) [10.1%]	3.4 (0.5) [10.8%]
BMI (kg/m2)	32.3 (6.4) [3.2%]	34.4 (6.5) [2.4%]	32.3 (6.4) [2.9%]	34.4 (6.6) [2.5%]
Outcome HbA1c (mmol/mol)	65.1 (16.0)	64.9 (14.2)	65.0 (16.2)	65.1 (14.6)
HbA1c_Month: Month of outcome HbA1c	9.2 (3.5)	9.0 (3.5)	9.2 (3.4)	9.0 (3.4)

### Prediction model for treatment selection based on HbA1c outcome

The model from Dennis et al. (2022) [2] assumes a continuous, normally distributed response variable $Y_{i}$ for $i=1,\dots ,n$, where *N* corresponds to the number of observations. The model is a standard linear regression model with both continuous and categorical predictors ($\textbf{X}^C_i$ and $\textbf{X}^D_i$, respectively). Restricted cubic splines are placed on the continuous predictors ($S\left( \textbf{X}^C_i\right)$) [36], and interaction terms are included between the binary variable for a drug taken ($X^T_i$) and the other variables. Hence the structure of the treatment selection model is:

3 $$\begin{aligned} Y_{i}{} & {} = \varvec{\beta }_0 + \varvec{\beta }_1 X^T_i + \varvec{\beta }_2 \textbf{X}_{i}^{D} + \varvec{\beta }_3 S\left( \textbf{X}_{i}^{C}\right) + \varvec{\beta }_4 X_{i}^{T}\textbf{X}_{i}^{D} + \varvec{\beta }_5 X_{i}^{T} S\left( \textbf{X}_{i}^{C}\right) + \epsilon _{i}\nonumber \\ \epsilon _i{} & {} \sim N\left( 0, \sigma ^2\right) , \end{aligned}$$

where $\varvec{\beta }_0$ is the intercept, $\varvec{\beta } = \left( \varvec{\beta }_1, \varvec{\beta }_2, \dots , \varvec{\beta }_5\right)$ is a vector of regression coefficients (where $\varvec{\beta }_2, \dots , \varvec{\beta }_5$ are themselves vectors of regression coefficients corresponding to the different discrete, spline and interactions variables as required), with $\sigma$ the residual standard deviation. Posterior summaries for the parameters can be found in Supplementary Table S1 and Fig. S5.

Hence, in the notation of the Study overview section, $Y_{i}$ and $X_{i}$ corresponds to the response and covariates for the individual *i*, such that $\textbf{Y} = \left( Y_1, \dots , Y_n\right)$ for the outcome, $\textbf{X} = \left( \textbf{X}_1, \dots , \textbf{X}_n\right)$ corresponds to the predictor values used in the modelling, where each observation’s covariates can be decomposed into $\textbf{X}_i = \left( \textbf{X}^D_i, \textbf{X}^C_i, X^T_i\right)$ and $\varvec{\psi } = (\varvec{\beta }_0, \varvec{\beta }, \sigma )$. For a full discussion of the form of this model, and the interpretation of the parameters, please see Dennis et al. (2022) [2].

### Model validation

To assess convergence and mixing of the MCMC chains, we perform a visual inspection of the trace plots for all parameters, as well as monitoring the Gelman-Rubin $\hat{R}$ values [37] (Supplementary Figs. S2–S3). The Gelman-Rubin $\hat{R}$ values for $\alpha$, $\sigma$ and regression parameters vary between 1 and 1.005. Together these diagnostics suggest that the chains converged and are mixing well. The model took 3 hours to fit with complete data and 24 hours to fit with incomplete data.

Since the application of DPMM in the model uses a truncated number of clusters, we also inspect the posterior mean number of individuals in components ranked by occupancy (Supplementary Fig. S2) alongside a posterior predictive plot of the fitted DPMM against the empirical distribution of the predictors from the development and validation data sets (Supplementary Figs. S4 and 1 respectively). Contrasting standard prediction models, the principal aspect of a treatment selection model is to accurately predict the optimal treatment instead of solely focusing on the outcome [38].

**Fig. 1:**
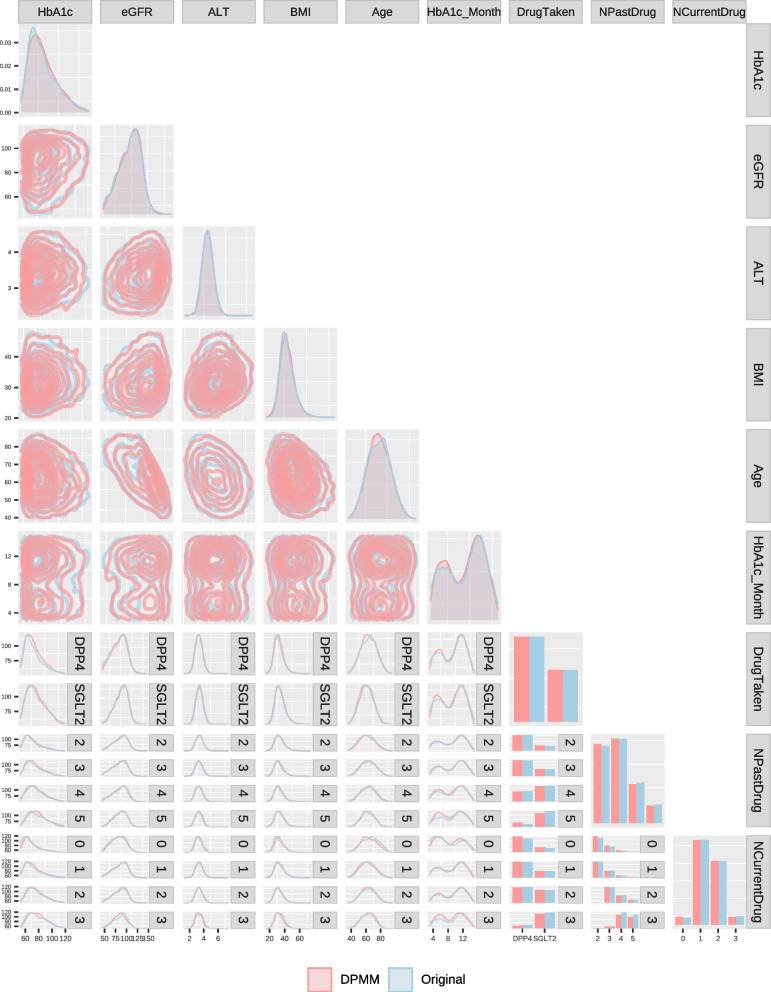
Generalised pairs plot of predictor variables for an independent validation dataset against an equal number of random samples from the Dirichlet process mixture model (DPMM). The DPMM provides an exceptional representation of the validation dataset

Validation of the individualised treatment effects of the model is carried out according to the guidelines provided by Dennis et al. [2]. The first method suggested is verifying the prediction performance by checking standardised residuals for predicted HbA1c—Supplementary Fig. S6. This shows that there is no major systematic bias in the mean function but that there is considerable residual variation as per the results of [2]. The second method is to plot a density graph for all patients regarding the difference between each therapy’s treatment effects (Supplementary Fig. S7) that can be compared to [2]. This shows the predicted number of patients benefiting from DPP4i or SGLT2i across the population and shows good agreement with [2].

## Results

### Fit and validation of the DPMM

To visualise the fit of the DPMM, we take the bivariate marginal posterior predictive distributions for a combination of variables versus the empirical equivalent (Fig. 1). The combination of density plots, density map plots and bar plots demonstrate that the fitted DPMM provides a good representation of both the development (Supplementary Fig. S4) and external validation (Fig. 1) datasets. We can see that the DPMM is very flexible and captures non-linearities, heteroscedasticity, correlation structures and mixtures of continuous and categorical variables.

### Probabilistic predictions using the Bayesian treatment selection model

**Fig. 2:**
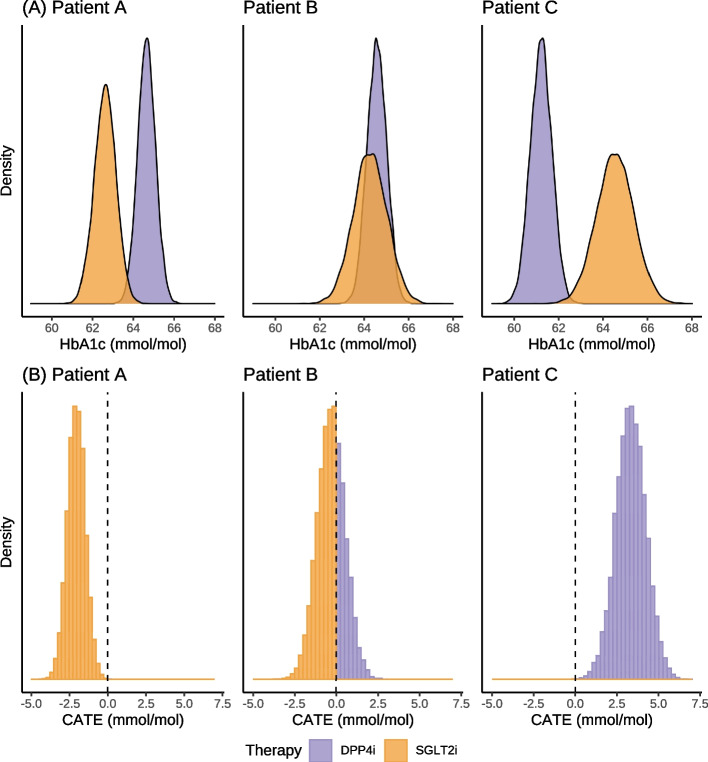
**A** Posterior predictive distributions for HbA1c outcome at 6-month post-treatment for three synthetic but representative patients. For patient A, SGLT2i has a 98% probability of performing better than DPP4i. **B** Predicted treatment response difference or conditional average treatment effect (CATE) at 6-months. A negative value corresponds to a benefit on SGLT2i, and a positive value corresponds to a benefit on DPP4i. For patient B, SGLT2i has a 58% probability of performing better than DPP4i. For patient C, DPP4i has a >99% probability of performing better than SGLT2i. Patient [A,B,C]: Number of Past Drugs [4,3,4], Number of Current Drugs [2,2,2], HbA1c [67,75,65], eGFR [84.2,66.6,67.9], ALT(log) [3.4,2.8,2.6], BMI [26.1,33.4,28.5], Age [68,79,81]

The Bayesian model produces full posterior predictive distributions that marginalise the uncertainty in the parameters and any missing predictor variables. This means we can make probabilistic statements about the parameter values and predicted outcomes based on the observed data and the choice of prior distributions. This is in contrast to frequentist approaches, where uncertainty statements relate to the expected long-term behaviour of an estimator if the same study were to be repeated *ad infinitum* [9]. In Fig. 2 (A), we showcase the posterior predictive distributions of average therapy response for three synthetic but representative patients (A, B and C) under the two competing treatments. An alternative way to visualise this information is to generate a posterior predictive distribution for the conditional average treatment effect (CATE) estimates (Fig. 2 (B)). Posterior samples for the CATE estimates can be calculated by subtracting the mean predicted treatment response of DPP4i from the mean predicted treatment response of SGLT2i (given the predictors) for each iteration of the fitted model. In this case, the proportion of the distribution to the left of the zero line corresponds to an estimate of the probability that SGLT2i is associated with a better average treatment response, and conversely, the proportion of the distribution to the right of the zero line is an estimate of the probability that DPP4i is associated with a better average treatment response. Hence the model predicts a 98% probability of SGLT2i performing better than DPP4i on average for patient A. For patient B, SGLT2i has a 58% probability of performing better on average than DPP4i, and for patient C the model predicts DPP4i as the optimal treatment on average with a >99% probability. With this approach, it is evident that there is a high probability of patient A having a 1–3 mmol/mol greater HbA1c reduction on average with SGLT2i instead of DPP4i. Patient B is predicted a possible average HbA1c benefit between 0–3 mmol/mol for both therapies. For patient C, there is a high probability of DPP4i resulting in a greater HbA1c reduction than SGLT2i, with a likely 1–7 mmol/mol benefit on average. As discussed earlier, these are posterior predictive distributions for the *mean* response for a patient with each set of characteristics (see Table 3).

**Table 3:** Posterior predictive distributions

In Bayesian modelling, it is possible to derive a full *posterior predictive distribution* for any new individual. Thus the uncertainty associated with a prediction is captured through a probability distribution, from which point estimates can be derived, or alternatively, probabilistic questions can be asked, such as those described in the Probabilistic predictions using the Bayesian treatment selection model section. The definition of a posterior predictive distribution for an individual with complete predictor information is:
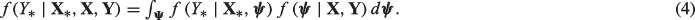
Thus, the predictive distribution *integrates* (or *averages*) over the posterior distribution for the parameters and thus naturally incorporates the uncertainties about the parameters as well as those arising from the underlying model. The examples in this paper present the posterior predictive distribution for the expected outcome, with a given set of characteristics defined by:
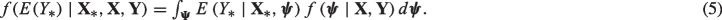
We are free to use either distribution (4–5), dependent on the context, although in practice, most treatment selection models use an analogous approach to (5), focusing on the conditional average treatment response. Please see the Supplementary Material for more information about how different predictive distributions can be derived and sampled from (including when predictors are missing, in which case we integrate the missing information modelled using the DPMM).

### Predictions for patients with missing data using the Bayesian treatment selection model

**Fig. 3:**
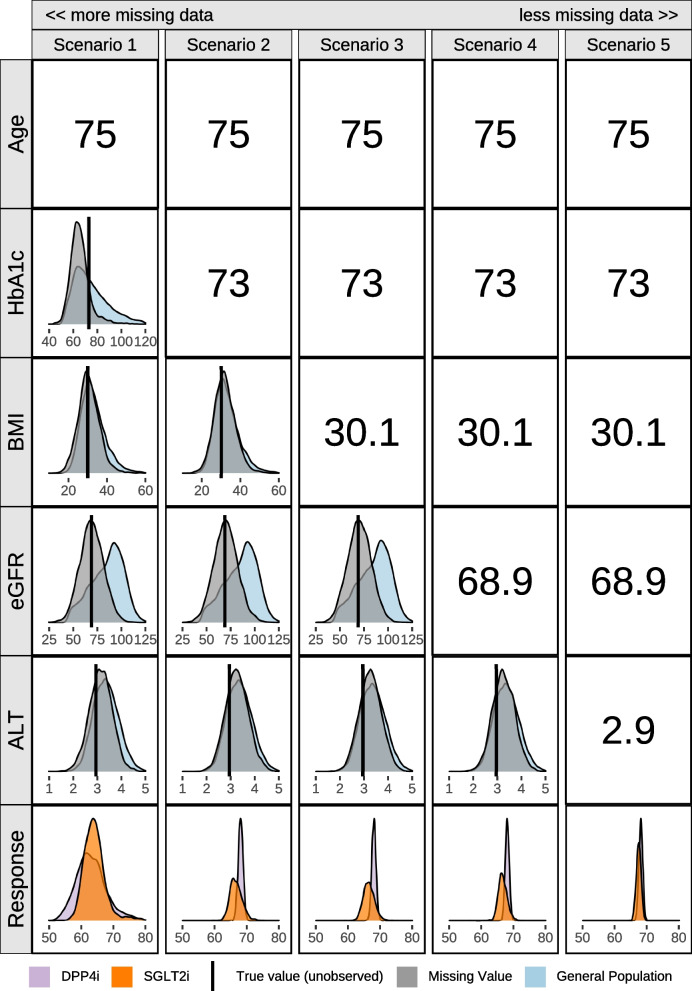
Predictive distributions for missing data and treatment response conditional on the scenario for a patient at 6-months. Unobserved true values are given by the black vertical line and its prediction by the grey distribution. As more variables become known, the uncertainty in the predictions decreases. Scenario 1: Past Drugs, Current Drugs, Age known. Scenario 2: Past Drugs, Current Drugs, Age, HbA1c known. Scenario 3: Past Drugs, Current Drugs, Age, HbA1c, BMI known. Scenario 4: Past Drugs, Current Drugs, Age, HbA1c, BMI, eGFR known. Scenario 5: All variables known. Patient: Number of Past Drugs [2], Number of Current Drugs [0], HbA1c [73], eGFR [68.9], ALT(log) [2.9], BMI [30.1], Age [75]

The inclusion of DPMM in the Bayesian model enables it to make predictions for patients with missing information. These predictions are possible by averaging over the conditional posterior predictive distributions for the missing values, given the existing data, and the uncertainty of the distributions is in part related to the degree of missingness. Figure 3 demonstrates how the data available for patient A influences the uncertainty of the posterior predictive distributions for the missing variables and the response. For each scenario, different sets of predictor variables are artificially missing. Each row displays how the uncertainty of the posterior predictive distributions for the missing covariates change as more data becomes available. The uncertainty of the posterior predictive treatment response distribution decreases as more variables become available. As baseline HbA1c is the most influential predictor in the model, the availability of the baseline HbA1c value vastly reduces the uncertainty of the prediction compared to other variables.

### Variable influence on the response prediction using the Bayesian treatment selection model

Since the model provides posterior predictive distributions for the missing data, a new question can be proposed—which missing variable will, if collected, give the most information about the treatment response? As an example, consider a patient with only three predictors available (number of previous therapies, current therapies and age). With these three predictors, the model predicts similar treatment responses for both DPP4i and SGLT2i. For each missing predictor, a range of treatment response distributions can be estimated for different values of the predictors, which have been chosen as different quantiles from the corresponding conditional posterior predictive distributions for the missing variables given the observed variables. This provides insight into how the treatment response prediction might be affected across a range of plausible predictor values.

**Fig. 4:**
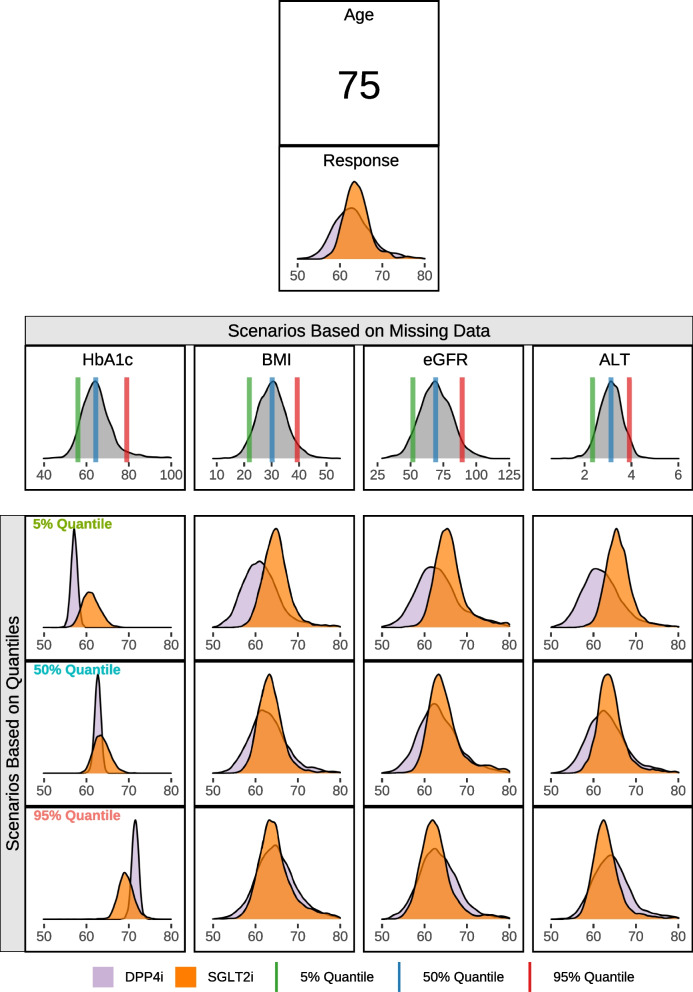
Analysis of variable influence on response prediction for a patient at 6-months. The initial response prediction is conditional on age, number of previous and current therapies. Further predictions are conditional on quantile values for HbA1c, BMI, eGFR and ALT. Out of the 4 missing covariates, the collection of HbA1c will, in theory, provide the most information about treatment response for the patient. Patient: Number of Past Drugs [2], Number of Current Drugs [0], Age [75]

Figure 4 showcases the conditional probability distributions for any missing variable, and we can explore what we might expect to see at different values informed by these distributions. For example, the treatment response changes drastically depending on starting HbA1c. For this patient, if HbA1c was equal to the 5% quantile value, there is a high probability the expected treatment response would be below 60 mmol/mol with DPP4i therapy but a much lower probability of being below the same threshold under SGLT2i. On the other hand, if HbA1c was equal to the 50% quantile value, then the expected HbA1c response is between 60–70 mmol/mol for both treatments, with considerable overlap between the treatment response predictions for both therapies. Lastly, if HbA1c was equal to the 95% quantile value, there is a low probability of the expected treatment response being below 70 mmol/mol for DPP4i but a higher probability of being below the same threshold for SGLT2i. In contrast, when other missing variables take different quantile values, it results in very similar treatment responses across all quantiles. All of the above provides evidence that, given the choice, starting HbA1c would be the most useful additional variable to collect to improve the choice of therapy for this patient.

### High-risk patient prediction using the Bayesian treatment selection model

**Fig. 5:**
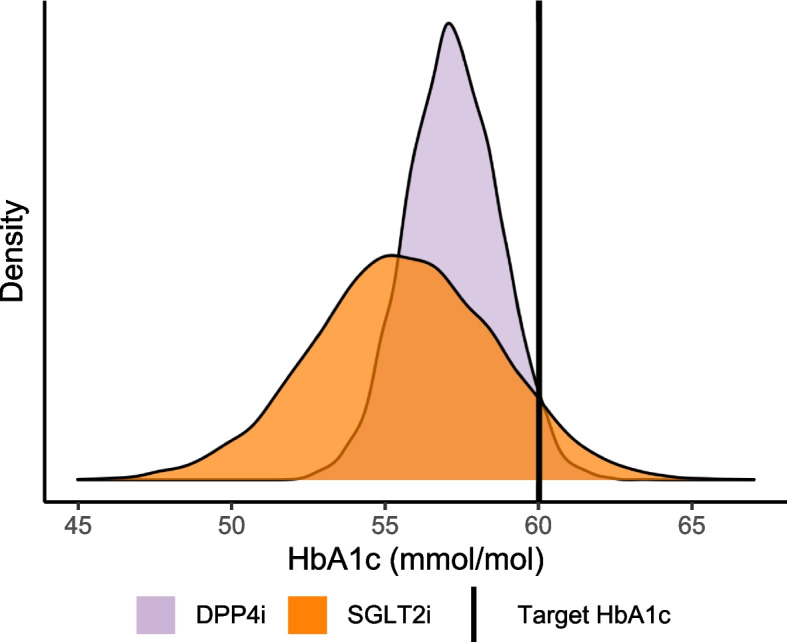
Therapy probability distribution at 6-month prediction for an arbitrary patient. The patient has a <60 mmol/mol target HbA1c outcome. In this situation, SGLT2i has a 7.7% chance of >60 mmol/mol treatment response, whereas DPP4i has a 3.1% chance of >60 mmol/mol. Due to the lower probability of treatment response above the target, DPP4i is the right therapy for this patient

In addition to all previous aspects, the Bayesian model could also be used in a different way. For example, in the case of a high-risk patient, it may be desirable to choose the treatment response that is most likely to result in a reduction of HbA1c to below 60 mmol/mol. Hence we could choose treatment on the basis of overall predicted benefit, or we could select the therapy with the highest probability of meeting some target threshold. Using Fig. 5 as a hypothetical example, there is a high probability of SGLT2i resulting in a better treatment response than DPP4i for this patient. In a conventional approach, SGLT2i would be the therapy chosen. However, it is critical to note that SGLT2i has a higher probability of resulting in a value of HbA1c above 60 mmol/mol than DPP4i. In this instance, the fact that DPP4i is less likely to result in a value above the target could be more influential in the choice of therapy than SGLT2i being more likely to result in lower treatment response on average. Choosing DPP4i as a therapy for this patient gives the highest probability of achieving an average treatment response below the target.

## Discussion

This paper provides details on how to fit a Bayesian treatment selection model with continuous and categorical covariates using a DPMM to capture the joint-covariate distribution. The model enables model fitting and response prediction for patients with complete and incomplete data (under the assumption that missingness is MCAR/MAR), similarly to [14, 27, 39]. It can be thought of as an alternative to Bayesian profile regression [11, 12] in which the outcome model-of-interest is conditioned explicitly on the covariates, rather than on the cluster memberships derived from the DPMM. This makes it more directly applicable to augment existing models. Crucially, it can be used to gain insight into the utility of collecting further data on patients with incomplete predictors in clinical practice.

The Bayesian treatment selection model presented in this paper augments the classical counterfactual model developed by Dennis et al. [2]. The model validation is carried out in an analogous way to the original model. This validation assesses whether the treatment selection model accurately predicts the optimal treatment rather than the exact therapy response. With that in mind, the performance of the Bayesian model is comparable to [2], even after augmenting the data to include the incomplete-case individuals (Supplementary Table S1 and Fig. S5). The regression parameter values are similar between the three models, with the Bayesian model achieving similar regularisation through the use of weakly informative regularising priors [40]. Including individuals with incomplete data in the model fitting process reduces the uncertainty of regression parameters marginally (Supplementary Fig. S5). This is because here the original data set was very large, and the number of individuals with incomplete data was a relatively small proportion of the data set (especially since most individuals would only be missing a few covariates at most). Hence, in this case, the missing data do not influence the posterior distributions for the parameters very much. However, this may not be true in other settings, and this approach allows a direct comparison of the difference between models fitted to complete-case data and incomplete-case data. More importantly, the treatment selection model now has a mechanism to produce predictions for new individuals with incomplete information, thus facilitating its use in clinical practice.

There are several additional benefits of this type of approach. In this case, we can estimate the treatment response alongside the difference in treatment response for both therapies. Furthermore, due to the inclusion of the DPMM, the model can provide predictive distributions for any missing covariates [14, 27, 39]. The availability of predictive distributions for missing covariates, in turn, can be used to influence the choice of future covariate data collection to improve treatment response prediction. Instead of having a set rule, we could use these distributions (Fig. 4) to choose to collect a variable that reduces the uncertainty in the predictive distribution for the difference between the two treatments. Alternatively, we could choose to collect a variable that optimises the posterior probability of preference for one therapy over the other. Moreover, since we obtain joint predictive distributions for all the missing variables, these can be utilised to look at the changes in expected treatment response for multiple variables simultaneously (although in Fig. 4 we show the univariate *marginal* predictive distributions for each variable, averaging over the others).

Furthermore, even in the presence of complete data, the inclusion of the DPMM in the model building enables the potential to make predictions for patients with incomplete predictor information, which is useful in the deployment of prediction models in clinical practice, where it may not be possible to collect all predictor information for a new patient. This is a novel approach with considerable advantages for prediction from treatment selection models, and many of these advantages would also be true for standard clinical prediction models. We have implemented this model in standard software (NIMBLE), and as such the DPMM can be used to augment any regression model supported by NIMBLE (for example, the same ideas could be applied to e.g. survival models, logistic regression and so on), without requiring new inference algorithms to be developed.

Currently, the assumption that missing predictor data are MCAR/MAR means that it is not necessary to explicitly model the missingness mechanism (as long as the parameters for the missingness model are *a priori* independent of the other parameters—in which case the missingness mechanism can be analytically integrated out, leaving the posterior defined in (2)). However, there are some restrictions. DPMMs, like many non-parametric approaches, are not good models for extrapolation. This means that for the DPMM to be a good representation of the joint distribution of the predictors, there needs to be some observed data throughout all regions of non-negligible density. As an extreme example, assume there are multiple predictors, $X_1, X_2, \dots , X_p$, where $X_1$ is *truncated*, based on $X_2$ (so $X_1$ is missing for any $X_2 > x^*$ say). Here the missingness mechanism for $X_1$ depends only on $X_2$, so the data are MAR. However, since there are no samples for $X_1$ in the region $X_2 > x^*$ the DPMM will be unable to adequately estimate the conditional distribution $f\left( X_1 \mid X_2 > x^*\right)$ on its own (although in hierarchical prediction models the response variable *Y* can also play a role in constraining the distributions for the missing data). In this case, we may need a more structured model for $X_1 \mid X_2$ if we wish to extrapolate into regions with no available data; although note that we can consider other hierarchical structures, such as $f\left( X_1 \mid X_2\right) f\left( X_2, \dots , X_p\right)$ where a DPMM is used to model the density $f\left( X_2, \dots , X_p\right)$. In this setting, despite major challenges (large sample size, large absolute numbers of missing information, reasonable numbers of covariates, clear non-normality in the data, combinations of continuous and categorical predictors) we were able to fit the described models in $\approx$24 hours. We note that this is substantially more than the complete-case model (3 hours), but it would be expected that the approach would be computationally challenging to fit for large datasets with lots of missing data and/or a high number of predictors. Despite this, once the models are fitted then predictions will remain relatively quick to obtain. On the other hand, a complete case analysis is straightforward to perform, only requiring the NIMBLE code we provide without the need for the custom MCMC samplers.

The further development of DPMM models is an active area of research, and some extensions that could be of interest in future work include exploring whether a more complex model structure, such as those previously discussed based on the joint modelling of the response and predictor variables [27] could improve predictive performance. The predictive performance could also be compared to simpler imputation techniques such as multivariate imputation by chained equations (MICE) (see [41] for more information). A key constraint in making these models straightforward and cost-effective to employ in clinical practice is ensuring that they are parsimonious, and hence this is an area where often variable selection methodology is employed, and although software exists to do this within DPMMs, such as the PReMiuM package in R [12], this currently only works for profile regression, or if a DPMM is used as a joint model for the response and predictors. There is published work exploring ways to handle missing data in prediction models when the missingness mechanism is MNAR (see e.g. [39, 42]), which may be relevant for other future applications. One potential advantage of separating the response model from the predictor distribution model is that cutting-edge treatment selection approaches such as Bayesian Causal Forests [43] could be used for the former, alongside a DPMM for the latter, but this would require more methodological development.

As a final note, we found that the DPMM structure employed here did a good job of capturing the covariate distribution without overfitting (Fig. 1 and Supplementary Fig. S4), but it is worth considering that there are many alternative ways to specify joint distributions with dependency structures amongst the predictors which may be applicable in other situations (see [44] for a review).

## Conclusion

In this work, we develop a hierarchical joint model using a spline-based treatment selection model for type 2 diabetes, alongside a DPMM as a flexible way of modelling the complex multivariate distribution of predictor variables. This facilitates both model inference and prediction in the presence of incomplete information. The inclusion of the DPMM in the joint model unlocks information that can be used by practitioners during the decision-making process. This modelling technique is being applied in treatment selection models for type 2 diabetes but could provide benefits to other types of prediction models.

### Supplementary Information

**Additional file 1.**

## Data Availability

The routine clinical data analysed during the current study are available in the CPRD repository (CPRD; https://cprd.com/research-applications), but restrictions apply to the availability of these data, which were used under license for the current study, and so are not publicly available. For re-using these data, an application must be made directly to CPRD. A synthetic sample data is available on GitHub within the repository “PM-Cardoso/DPMM-tsm”.
