# Supplementary material for: Dirichlet process mixture models to impute missing predictor data in counterfactual prediction models: an application to predict optimal type 2 diabetes therapy
Source: BMC Med Inform Decis Mak. 2024 Jan 8;24:12. doi: 10.1186/s12911-023-02400-3 (PMC10773072; doi:10.1186/s12911-023-02400-3)
Supplement: Supplementary file 1 — Additional file 1. [file 12911_2023_2400_MOESM1_ESM.pdf]

Supplementary material: Dirichlet process mixture models to impute missing predictor data in counterfactual prediction models: an application to predict optimal type 2 diabetes therapy

Pedro Cardoso[1], John M. Dennis[1], Jack Bowden[1], Beverley M. Shields[1],
Trevelyan J. McKinley[†1] and the MASTERMIND Consortium[*]

[†]Corresponding Author, t.mckinley@exeter.ac.uk

[1]University of Exeter, Medical School, Exeter, England

[*]A list of authors and their affiliations appears at the end of the paper

S1 Appendix

S1.1 Component distributions

As described in Box 1, for J_C continuous predictors, we use mixtures of multivariate Gaussian distributions with J_C dimensions, with cluster specific parameters for component k ($k = 1, \ldots, K$) given by $(\boldsymbol{\mu}_k, \boldsymbol{\Sigma}_k)$, where $\boldsymbol{\mu}_k$ is a J_C-vector of means and $\boldsymbol{\Sigma}_k$ is a $(J_C \times J_C)$ covariance matrix. We use a latent variable Z_i to denote the cluster membership for individual i, and hence the conditional component density given Z_i is:

$$f_{Z_i}\left(\mathbf{X}_i^C \mid \boldsymbol{\mu}_{Z_i}, \boldsymbol{\Sigma}_{Z_i}\right) = \frac{1}{\sqrt{(2\pi)^J \mid \boldsymbol{\Sigma}_{Z_i} \mid}} \exp\left[-\frac{1}{2}\left(\mathbf{X}_i^C - \boldsymbol{\mu}_{Z_i}\right)^T \boldsymbol{\Sigma}_{Z_i}^{-1}\left(\mathbf{X}_i^C - \boldsymbol{\mu}_{Z_i}\right)\right]. \tag{S.1}$$

For J_D categorical predictors, we use mixtures of categorical probability mass functions, where the number of categories for a covariate j ($j = 1, \ldots, J_D$) is K_j and the component-specific parameters are the probabilities of belonging to each category, given by $\boldsymbol{\phi}_k = (\boldsymbol{\phi}_{k1}, \boldsymbol{\phi}_{k2}, ..., \boldsymbol{\phi}_{kJ_D})$ with $\boldsymbol{\phi}_{kj} = (\phi_{kj1}, \phi_{kj2}, \ldots, \phi_{kjK_j})$ and $\sum_{l=1}^{K_j} \phi_{kjl} = 1$. Hence

$$f_{Z_i}(\mathbf{X}_i^D \mid \boldsymbol{\phi}_{Z_i}) = \prod_{j=1}^{J} \boldsymbol{\phi}_{Z_i j}^{X_{ij}}. \tag{S.2}$$

S1.2 Prior distributions

We use a stick-breaking [1, 2] construction for the prior probabilities of the mixture components p_k. Conceptually, this involves repeatedly breaking off and discarding a random fraction of a stick with an initial length

of 1. The fraction discarded is sampled from a Beta distribution with shape parameter α (where α influences the prior weights on the number of components).

$$\begin{aligned}
p_k &= V_k \prod_{l<k}(1 - V_l) \\
p_1 &= V_1 \\
V_k &\sim \text{Beta}(1, \alpha)
\end{aligned}$$

In order to improve convergence and mixing of the MCMC algorithm, all continuous variables are standardised. The regression parameters ($\boldsymbol{\beta}$) are fitted with weakly informative shrinkage priors, which acts as a regularisation technique and can reduce posterior uncertainty alongside stabilising computations [3]. The prior distributions for regression parameters and σ are:

$$\begin{aligned}
\boldsymbol{\beta} &\sim \text{Normal}(0, 2.5) \\
\sigma &\sim \text{Exponential}(1)
\end{aligned}$$

The clustering and components of the DPMM follows a hierarchical structure, with the following prior distributions:

$$\begin{aligned}
Z_i &\sim \text{Categorical}(k, \pi) \\
\pi &\sim \text{Beta}(1, \alpha) \\
\alpha &\sim \text{Gamma}(\text{shape} = 2, \text{rate} = 1) \\
\boldsymbol{\mu}_k &\sim \text{Multivariate Normal}(\boldsymbol{\mu}_0, \boldsymbol{\Sigma}_0) \\
\boldsymbol{\Sigma}_k^{-1} &\sim \text{Wishart}(\text{R}, \rho) \\
R &\sim \text{Wishart}(\text{R}_0, \rho_0) \\
\rho &\sim \text{Exponential}(0.1)
\end{aligned}$$

We set $\boldsymbol{\mu}_0$ to be a vector of means of continuous variables and we set $\boldsymbol{\Sigma}_0$ to be the covariance matrix corresponding to a diagonal matrix where the diagonal entries are equal to the magnitude of the range of each continuous covariate. For R, we set the degrees of freedom to the number of continuous variables in the model, $\rho_0 = 6$. Although we achieve a good fit with $\alpha \sim \text{Gamma}(\text{shape} = 2, \text{rate} = 1)$, this may not hold for other applied cases, and other ways of defining this hyperparameter could be considered. See Liverani *et al.*, 2015 for a justification of these choices [4].

S1.3 Predictive distributions

The Bayesian hierarchical model has posterior distribution:

$$f(\boldsymbol{\psi}, \boldsymbol{\Theta} \mid \mathbf{X}, \mathbf{Y}) \propto f(\mathbf{Y} \mid \mathbf{X}, \boldsymbol{\psi}) f(\mathbf{X} \mid \boldsymbol{\Theta}) f(\boldsymbol{\psi}, \boldsymbol{\Theta}). \tag{S.3}$$

If there are no missing predictor variables, then the DPMM provides no additional information about the treatment selection model parameters, and could be integrated out of (S.3). The purpose of including the DPMM is two-fold: a) to allow incomplete predictor data to be included when the model is fitted; and b) to allow predictions to be made for individuals with missing predictor information. Hence, if we have a combination of missing ($\mathbf{X}^m$) and non-missing ($\mathbf{X}^o$) predictors, then the target posterior distribution is:

$$f(\boldsymbol{\psi}, \boldsymbol{\Theta}, \mathbf{X}^m \mid \mathbf{X}^o, \mathbf{Y}) \propto f(\mathbf{Y} \mid \mathbf{X}^o, \mathbf{X}^m, \boldsymbol{\psi}) f(\mathbf{X}^o, \mathbf{X}^m \mid \boldsymbol{\Theta}) f(\boldsymbol{\psi}, \boldsymbol{\Theta}), \tag{S.4}$$

and as such we can obtain full posterior predictive distributions for all of the missing variables $\mathbf{X}^m$ in addition to the model parameters. We can then integrate over the missing variables to get the marginal posterior distribution for the parameters-of-interest:

$$f(\boldsymbol{\psi}, \boldsymbol{\Theta} \mid \mathbf{X}^o, \mathbf{Y}) = \int_{\mathbf{X}^m} f(\boldsymbol{\psi}, \boldsymbol{\Theta}, \mathbf{X}^m \mid \mathbf{X}, \mathbf{Y}) d\mathbf{X}^m. \tag{S.5}$$

This (multi-dimensional) integral can be done numerically using the posterior samples generated from the MCMC. Thus the Bayesian model naturally propagates the uncertainties from the missing information through to the posterior distributions for the parameters.

Similarly, since the DPMM provides a flexible joint probability model for the predictor variables, we can leverage this to produce posterior predictive distributions for a new individual with observed predictors $\mathbf{X}_*^o$ say. In this case

$$f(Y_*, \mathbf{X}_*^m \mid \mathbf{X}_*^o, \mathbf{X}^o, \mathbf{Y}) = \int_{\boldsymbol{\Theta}} \int_{\boldsymbol{\psi}} f\left(Y_* \mid \mathbf{X}_*^m, \mathbf{X}_*^o, \boldsymbol{\psi}\right) f\left(\mathbf{X}_*^m \mid \mathbf{X}_*^o, \boldsymbol{\Theta}\right) f\left(\boldsymbol{\psi}, \boldsymbol{\Theta} \mid \mathbf{X}^o, \mathbf{Y}\right) d\boldsymbol{\psi} d\boldsymbol{\Theta}, \tag{S.6}$$

gives the joint posterior predictive distribution for Y_* and $\mathbf{X}_*^m$, where $f\left(\mathbf{X}_*^m \mid \mathbf{X}_*^o, \boldsymbol{\Theta}\right)$ is the conditional distribution for $\mathbf{X}_*^m$ given $\mathbf{X}_*^o$, which can be derived directly from the DPMM (see Section S1.4 for details). Again, these distributions can be estimated via Monte Carlo simulation, using the posterior samples generated from the MCMC, and then simulating from the conditional DPMM and the treatment selection model for each set of samples. Estimates of the marginal posterior predictive distributions for $f(Y_* \mid \mathbf{X}_*^o, \mathbf{X}^o, \mathbf{Y}, \boldsymbol{\psi})$ and $f(X_*^m \mid \mathbf{X}_*^o, \mathbf{X}^o, \mathbf{Y}, \boldsymbol{\Theta})$ can be readily generated in a similar way.

In the case where a new individual has complete covariate information, then the posterior predictive

distribution for Y_* reduces to

$$f(Y_* \mid \mathbf{X}_*, \mathbf{X}^o, \mathbf{Y}) = \int_{\boldsymbol{\psi}} f\left(Y_* \mid \mathbf{X}_*, \boldsymbol{\psi}\right) f\left(\boldsymbol{\psi} \mid \mathbf{X}^o, \mathbf{Y}\right) d\boldsymbol{\psi}, \tag{S.7}$$

and the DPMM parameters analytically integrate out. Following Dennis *et al.* [5], the plots presented in this paper ignore the residual variation and generate posterior distributions for expected responses. See Box 2 for more information. Full details are given in Section S1.4 and example code for fitting the model, and generating posterior predictive samples is given as additional supplementary material.

S1.4 Conditional predictive sampling

Posterior predictive distributions for patients with missing information can be generated empirically using posterior samples generated from the MCMC, as long as we can sample from a conditional DPMM and the treatment selection model (both described in Section S1.3). Suppose $\mathbf{X}_*$ is a J-dimensional vector of covariates for a new individual. We partition $\mathbf{X}_*$ into two disjoint subsets $\mathbf{X}_*^m$ and $\mathbf{X}_*^o$, where $\mathbf{X}_*^m$ are the missing covariates of M dimensions and $\mathbf{X}_*^o$ are the observed covariates of $J-M$ dimensions. We will estimate the joint posterior predictive distribution for the response Y_* and $\mathbf{X}_*^m$ given the observations $\mathbf{X}_*^o$:

$$f\left(Y_*, \mathbf{X}_*^m \mid \mathbf{X}_*^o, \mathbf{X}_{1:N}, \mathbf{Y}_{1:N}\right) = \int_{\boldsymbol{\psi}} \int_{\boldsymbol{\Theta}} f\left(Y_* \mid \mathbf{X}_*^m, \mathbf{X}_*^o, \boldsymbol{\psi}\right) f\left(\mathbf{X}_*^m \mid \mathbf{X}_*^o, \boldsymbol{\Theta}\right) f\left(\boldsymbol{\psi}, \boldsymbol{\Theta} \mid \mathbf{Y}_{1:N}, \mathbf{X}_{1:N}^o\right) d\boldsymbol{\Theta} d\boldsymbol{\psi}, \tag{S.8}$$

where $f\left(\boldsymbol{\psi}, \boldsymbol{\Theta} \mid \mathbf{Y}_{1:N}, \mathbf{X}_{1:N}^o\right)$ is the marginal posterior distribution given the observed data $\mathbf{Y}_{1:N}$ and $\mathbf{X}_{1:N}^o$. The probability density for individual i, given Z_i is:

$$f(\mathbf{X}_i \mid Z_i, \boldsymbol{\Theta}_{Z_i}) = f_{Z_i}\left(\mathbf{X_i} \mid \boldsymbol{\Theta}_{Z_i}\right) = f(\mathbf{X}_i^C \mid \boldsymbol{\mu}_{Z_i}, \boldsymbol{\Sigma}_{Z_i}) f(\mathbf{X}_i^D \mid \boldsymbol{\phi}_{Z_i}) \tag{S.9}$$

We can estimate (S.8) through Monte Carlo sampling, by first drawing L random samples, $(\boldsymbol{\psi}_l, \boldsymbol{\Theta}_l)$ $(l = 1, \dots, L)$, from the posterior distribution (obtained through the original MCMC runs), and then for each of these we sample from $f\left(\mathbf{X}_{*l}^m \mid \mathbf{X}_*^o, \boldsymbol{\Theta}_l\right)$ and then $f\left(\mathbf{Y}_{*l} \mid \mathbf{X}_{*l}^m, \mathbf{X}_*^o, \boldsymbol{\psi}_l\right)$ as detailed below.

For a given set of parameters $\boldsymbol{\Theta}$, we need to generate random samples from:

$$f\left(\mathbf{X}_*^m \mid \mathbf{X}_*^o, \boldsymbol{\Theta}\right) = \sum_{z=1}^{K} f\left(\mathbf{X}_*^m \mid Z = z, \mathbf{X}_*^o, \boldsymbol{\Theta}\right) P\left(Z = z \mid \mathbf{X}_*^o, \boldsymbol{\Theta}\right). \tag{S.10}$$

We can sample from (S.10) by first drawing a component Z from:

$$\begin{aligned} P\left(Z = z \mid \mathbf{X}_*^o, \boldsymbol{\Theta}\right) &\propto f\left(\mathbf{X}_*^o \mid Z = z, \boldsymbol{\Theta}\right) f\left(Z = z \mid \boldsymbol{\Theta}\right) && \text{from Bayes' Theorem} \\ &\propto f_z\left(\mathbf{X}_*^{Co} \mid \boldsymbol{\mu}_z, \boldsymbol{\Sigma}_z\right) f_z\left(\mathbf{X}_*^{Do} \mid \boldsymbol{\phi}_z\right) f\left(Z = z \mid \boldsymbol{\pi}, \alpha\right) && \text{from (S.9)}, \end{aligned}$$

where $\mathbf{X}_*^{Co}$ and $\mathbf{X}_*^{Do}$ are the observed continuous and categorical variables respectively. Then, given $Z = z$, we have

$$\begin{aligned} f\left(\mathbf{X}_*^m \mid Z = z, \mathbf{X}_*^o, \boldsymbol{\Theta}\right) &= f\left(\mathbf{X}_*^{Cm} \mid Z = z, \mathbf{X}_*^o, \boldsymbol{\Theta}\right) f\left(\mathbf{X}_*^{Dm} \mid Z = z, \mathbf{X}_*^o, \boldsymbol{\Theta}\right) \\ &= f_z\left(\mathbf{X}_*^{Cm} \mid \mathbf{X}_*^{Co}, \boldsymbol{\mu}_z, \boldsymbol{\Sigma}_z\right) f_z\left(\mathbf{X}_*^{Dm} \mid \mathbf{X}_*^{Do}, \boldsymbol{\phi}_z\right) \quad \text{from (S.9).} \end{aligned} \tag{S.11}$$

We can sample from (S.11) by taking independent random samples for $\left(\mathbf{X}_*^{Cm} \mid \mathbf{X}_*^{Co}, \boldsymbol{\mu}_z, \boldsymbol{\Sigma}_z\right)$ and $\left(\mathbf{X}_*^{Dm} \mid \mathbf{X}_*^{Do}, \boldsymbol{\phi}_z\right)$, where

$$\left(\mathbf{X}_*^{Cm} \mid \mathbf{X}_*^{Co}, \boldsymbol{\mu}_z, \boldsymbol{\Sigma}_z\right) \sim N\left(\boldsymbol{\mu}_{*z}^{m|o}, \boldsymbol{\Sigma}_z^{m|o}\right),$$

with

$$\begin{aligned} \boldsymbol{\mu}_{*z}^{m|o} &= \boldsymbol{\mu}_z^m + \boldsymbol{\Sigma}_z^{mo}\left(\boldsymbol{\Sigma}_z^{oo}\right)^{-1}\left(\mathbf{X}_*^{Co} - \boldsymbol{\mu}_z^o\right) \\ \boldsymbol{\Sigma}_z^{m|o} &= \boldsymbol{\Sigma}_z^{mm} - \boldsymbol{\Sigma}_z^{mo}\left(\boldsymbol{\Sigma}_z^{oo}\right)^{-1}\boldsymbol{\Sigma}_z^{om}, \end{aligned}$$

assuming that $\boldsymbol{\mu}_z^C$ and $\boldsymbol{\Sigma}_z^C$ are partitioned such that

$$\boldsymbol{\mu}_z^C = \begin{pmatrix} \boldsymbol{\mu}_z^m \\ \boldsymbol{\mu}_z^o \end{pmatrix} \quad \text{and} \quad \boldsymbol{\Sigma}_z^C = \begin{pmatrix} \boldsymbol{\Sigma}_z^{mm} & \boldsymbol{\Sigma}_z^{mo} \\ \boldsymbol{\Sigma}_z^{om} & \boldsymbol{\Sigma}_z^{oo} \end{pmatrix}.$$

Finally, we sample

$$\left(\mathbf{X}_{*j}^{Dm} \mid \boldsymbol{\phi}_z\right) \sim \text{Multinomial}\left(\boldsymbol{\phi}_{kj}\right)$$

independently for $j = 1, \ldots, M$ missing covariates (since $\mathbf{X}_{*j}^{Dm}$ are conditionally independent of $\mathbf{X}_{*j}^{Do}$ given cluster $Z = z$).

Once we have L random samples for $\mathbf{X}_{*l}^m$, we can then sample (with a slight abuse of notation) from the treatment selection model such that $Y_{*l} \sim f\left(Y \mid \mathbf{X}_{*l}^m, \mathbf{X}_*^o, \boldsymbol{\psi}_l\right)$.

S1.5 Convergence diagnostics

The Bayesian model was run for 50 000 iterations in two separate chains. Inspection of α values (Figure S2C) as well as σ and regression parameters (Figure S3) revealed that the first twenty thousand iterations should be discarded as burn-in. The trace plots for the regression parameters demonstrate the remaining iterations after the burn-in suggest convergence of the model (Figure S3). During the model fit, not all components had patients assigned to them. After removing burn-in and ranking components by occupancy (Figure S2B), only 18 components were utilised with components below the 14th rank having less than 30 patients (0.2%) assigned to them (Figure S2A). The Gelman-Rubin $\hat{R}$ values for α, σ and regression parameters vary between

1 and 1.005.

S1.6 The Bayesian treatment selection model is consistent with the original penalised maximum likelihood regression model

The posterior distributions for the regression parameters in the model fitted to incomplete data stay consistent with the equivalent model fitted to complete data in both Bayesian and maximum likelihood approaches (Figure S5). The posterior credible intervals for the Bayesian model fitted to the complete data are slightly narrower on the whole than the frequentist confidence intervals, due to the weak shrinkage priors used. We can see that the inclusion of the incomplete data results in a reduction of the posterior intervals, due to an increase in the number of data points available to inform the model fit.

Internal validation of the model shows the final model explained 29% of the variation of HbA1c outcome, with a good calibration (slope = 1.0015 [1 = perfect]) (Figure S6A). Validation of the model in the hold-out dataset shows that the model explained 29% of the variation of the HbA1c outcome, alongside a good calibration (slope = 1.02) (Figure S6B). In the development dataset, 13368 patients are predicted to benefit better from SGLT2i therapy, and 2758 patients are predicted to benefit better from DPP4i therapy. In cases where SGLT2i is predicted as the optimal therapy, 176 patients are predicted a benefit >10 mmol/mol on average and 6355 patients are predicted to benefit between 5–10 mmol/mol on average. Whereas when DPP4i is predicted as the optimal therapy, 316 patients are predicted a benefit >5 mmol/mol (Figure S7A) on average. In the validation dataset, 8929 patients are predicted to benefit from SGLT2i therapy, and 1822 patients are predicted to benefit from DPP4i therapy. In cases where SGLT2i is predicted as the optimal therapy, 123 patients are predicted a benefit > 10 mmol/mol on average and 4198 patients are predicted to benefit between 5–10 mmol/mol on average. Compared with when DPP4i is predicted as the optimal therapy, 209 patients are predicted a benefit >5 mmol/mol (Figure S7B) on average.

S2 Supplementary Tables

Model Parameters	Mean	Lower CI	Higher CI
Intercept	0.69	0.57	0.80
HbA1c	0.58	0.53	0.63
eGFR	0.02	-0.02	0.06
ALT	-0.02	-0.06	0.02
BMI	0.04	-0.004	0.09
Age	-0.06	-0.11	-0.01
HbA1c_Month	-0.04	-0.05	-0.03
Drug Taken	-0.61	-0.50	-0.39
Number of Past Drugs [1]	0.28	0.24	0.32
Number of Past Drugs [2]	0.51	0.45	0.56
Number of Past Drugs [3]	0.64	0.57	0.71
Number of Current Drugs [1]	-0.40	-0.46	-0.33
Number of Current Drugs [2]	-0.55	-0.63	-0.48
Number of Current Drugs [3]	-0.66	-0.76	-0.56
Spline(HbA1c)	-0.11	-0.18	-0.03
Spline(eGFR)	-0.0002	-0.04	0.04
Spline(ALT)	0.02	-0.03	0.07
Spline(BMI)	-0.02	-0.08	0.04
Spline(Age)	-0.05	-0.10	0.01
Spline(HbA1c_Month)	0.02	0.01	0.03
Drug Taken * HbA1c	-0.26	-0.35	-0.17
Drug Taken * eGFR	-0.12	-0.20	-0.04
Drug Taken * ALT	-0.09	-0.16	-0.02
Drug Taken * BMI	-0.12	-0.20	-0.04
Drug Taken * Age	0.04	-0.04	0.12
Drug Taken * Spline(HbA1c)	0.26	0.13	0.39
Drug Taken * Spline(eGFR)	0.04	-0.03	0.12
Drug Taken * Spline(ALT)	0.02	-0.05	0.10
Drug Taken * Spline(BMI)	0.12	0.02	0.22
Drug Taken * Spline(Age)	0.05	-0.05	0.15

Supplementary Table S1: Model parameter Bayesian posterior samples fitted for the incomplete datasets.

S3 Supplementary Figures

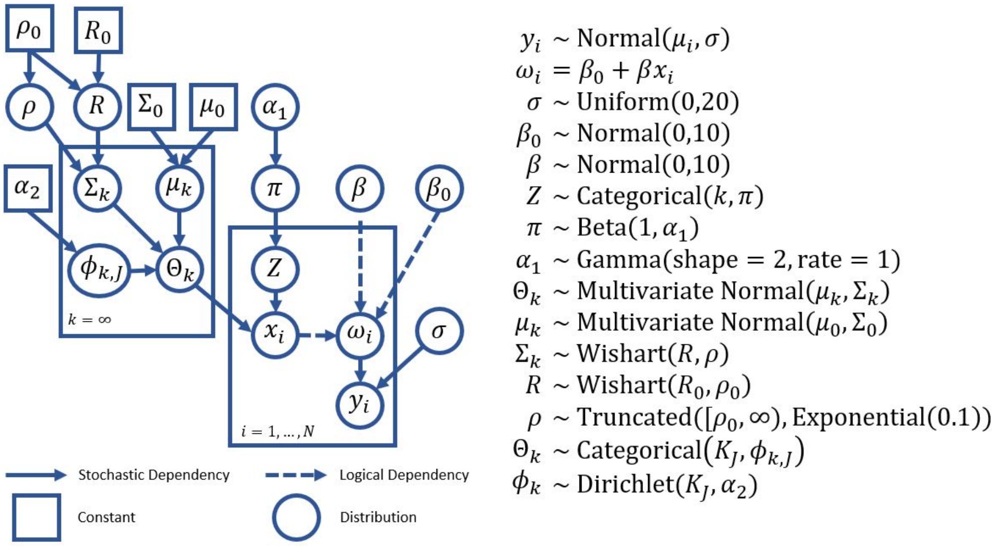

Supplementary Figure S1: A Directed Acyclic Graph (DAG) of the Bayesian treatment selection model $(y_i, \omega_i, \sigma, \boldsymbol{\beta}, \boldsymbol{\beta_0}, \mathbf{x}_i)$ augmented with a Dirichlet Process Mixture Model (DPMM). The DPMM component k assigned for each patient Z_i, is defined by π and $\alpha_{\mathbf{1}}$. The DPMM is given by a mixture of Gaussian and discrete variables. For Gaussian mixtures, the component specific parameters are $\boldsymbol{\mu}_k$ and $\boldsymbol{\Sigma}_k$, with other parameters as priors (μ_0,Σ_0,R,ρ,R_0,ρ_0). For discrete mixtures, the component specific parameters is $\boldsymbol{\phi}_{kJ}$ with a flat Dirichlet prior for K_J variable categories.

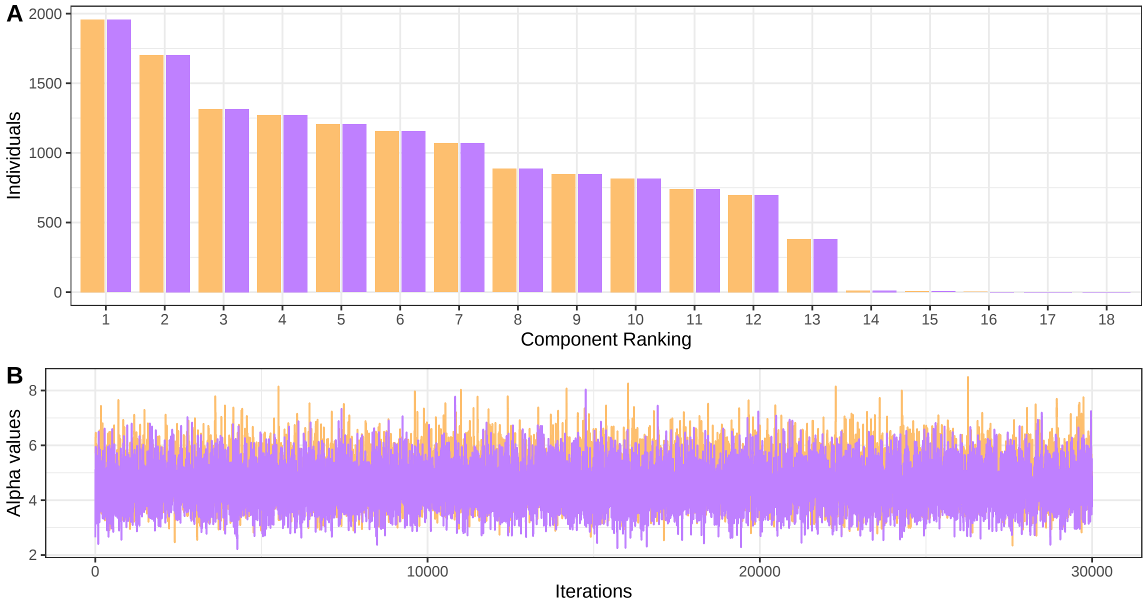

Supplementary Figure S2: A: Average number of patients in components ranked by numbers of membership at each iteration after convergence. B: Trace plots of the Dirichlet process mixture model (DPMM) alpha parameter and number of components utilised for 2 chains with 30,000 iterations (20,000 iterations of burn-in removed).

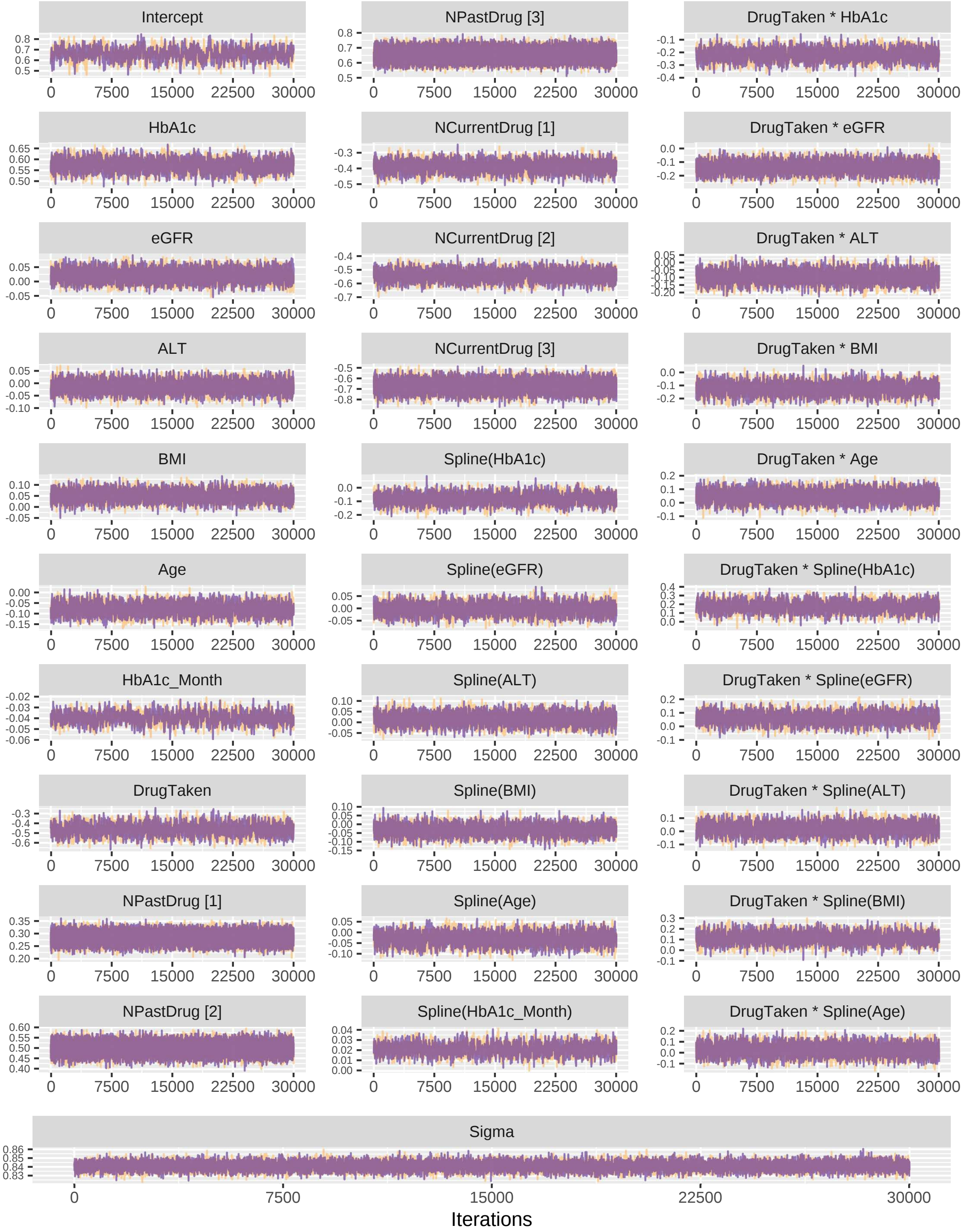

Supplementary Figure S3: Trace plots of regression parameters for two chains each with 30,000 iterations (20,000 iterations of burn-in removed).

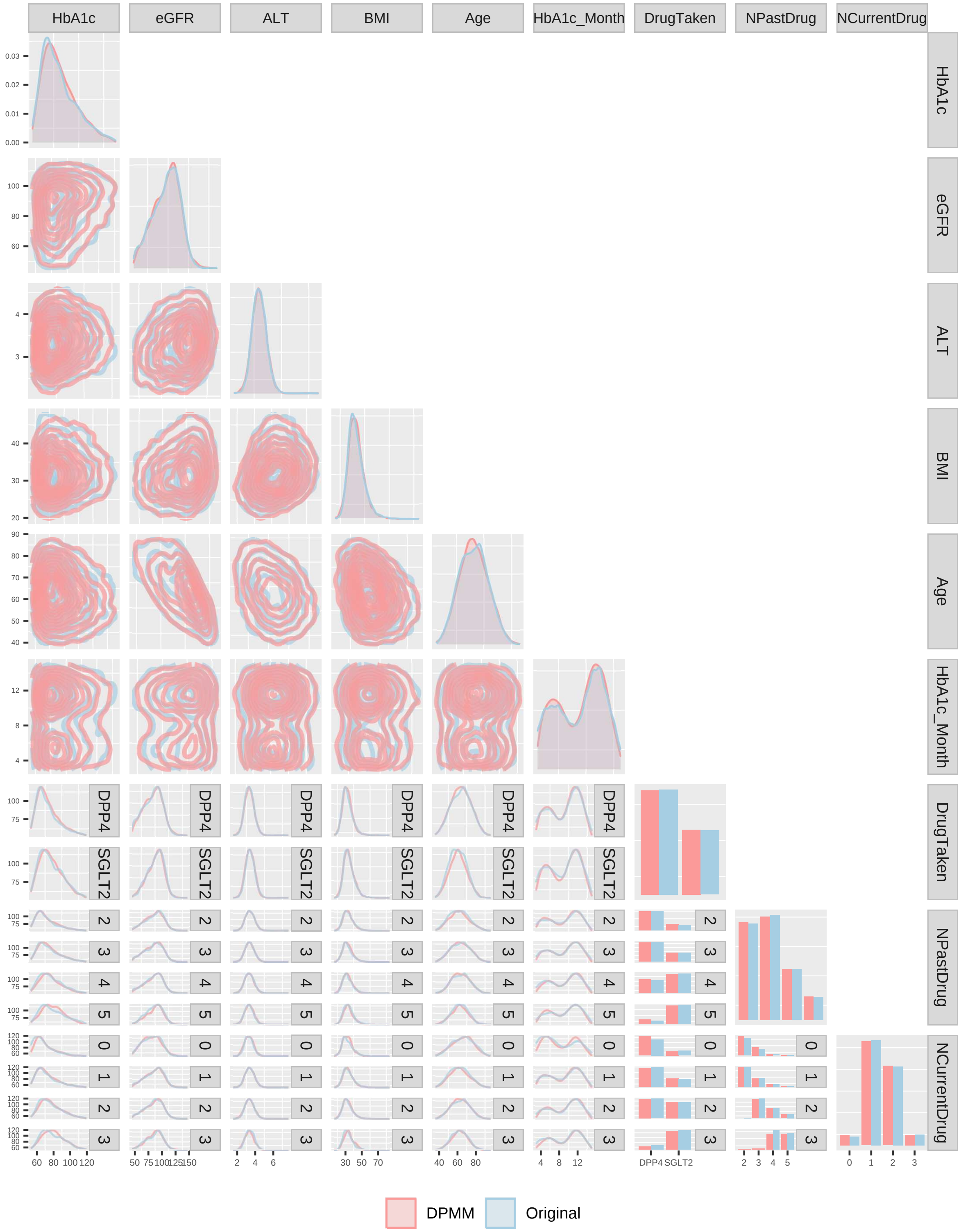

Supplementary Figure S4: Generalised pairs plot of predictor variables for the development dataset against an equal number of posterior predictive samples from the Dirichlet process mixture model (DPMM).

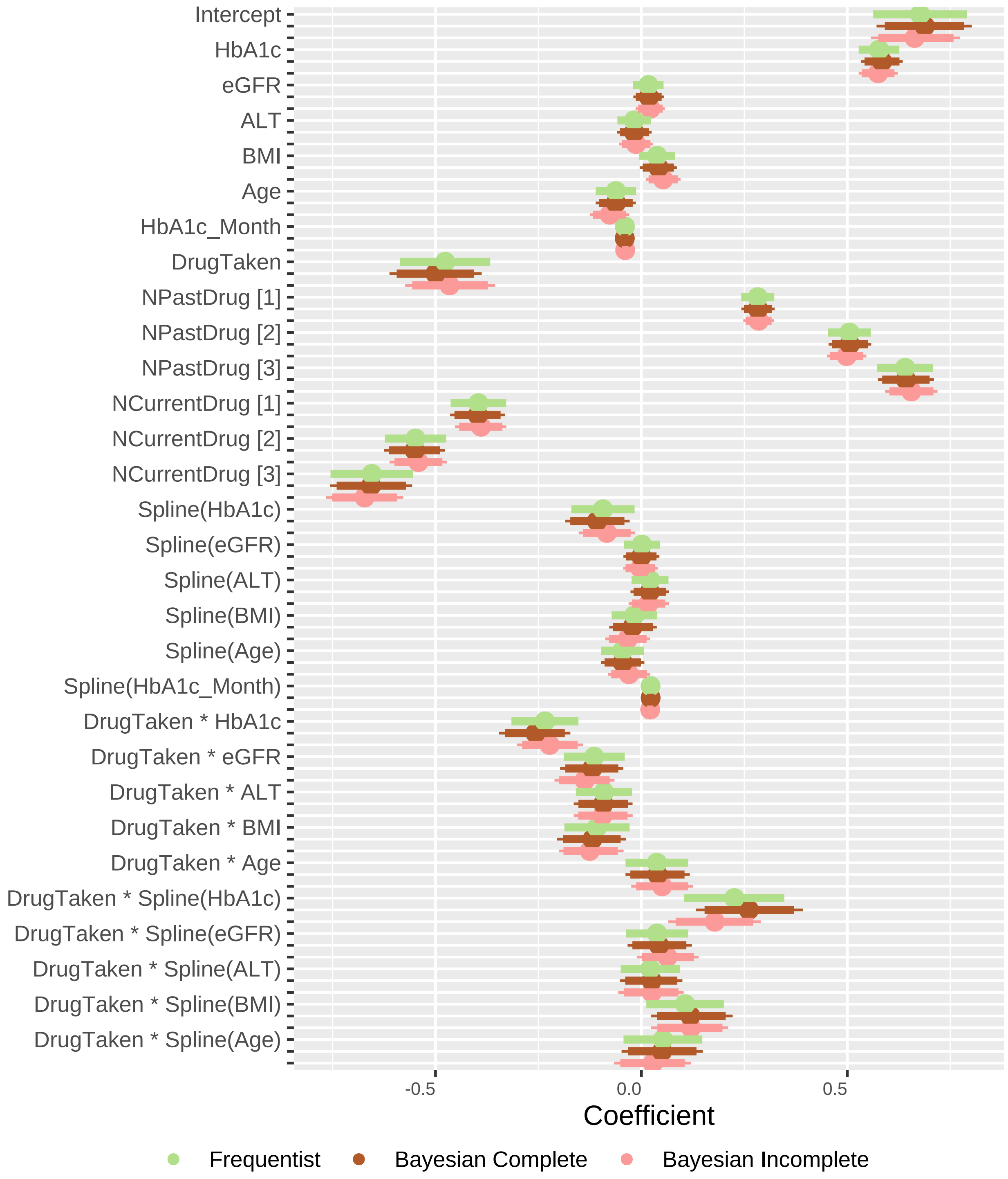

Supplementary Figure S5: Caterpillar plot comparing Bayesian posterior samples fitted for the complete and incomplete datasets against the frequentist coefficient estimates. For each set of Bayesian posterior samples, the plot shows the 2.5%, 5%, 50%, 95% and 97.5% quantiles. For the frequentist estimates, the plot displays the 95% confidence interval.

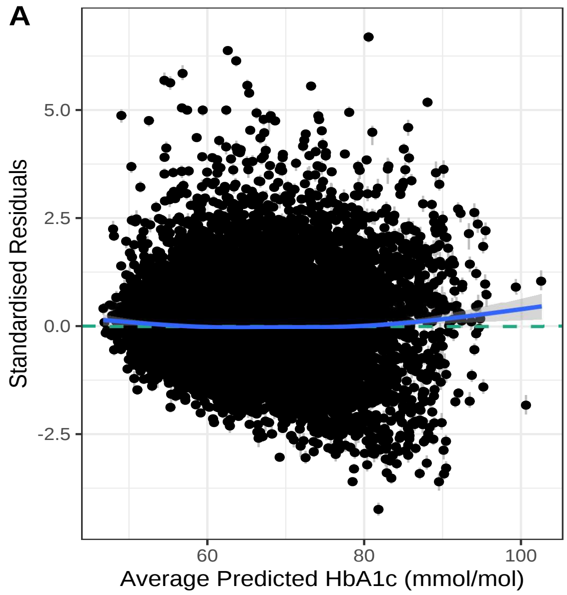

Supplementary Figure S6: Standardised residuals of predicted outcome (at 6 months) for development (A) and validation (B) datasets, utilising posterior predictions from the fitted Bayesian treatment selection model.

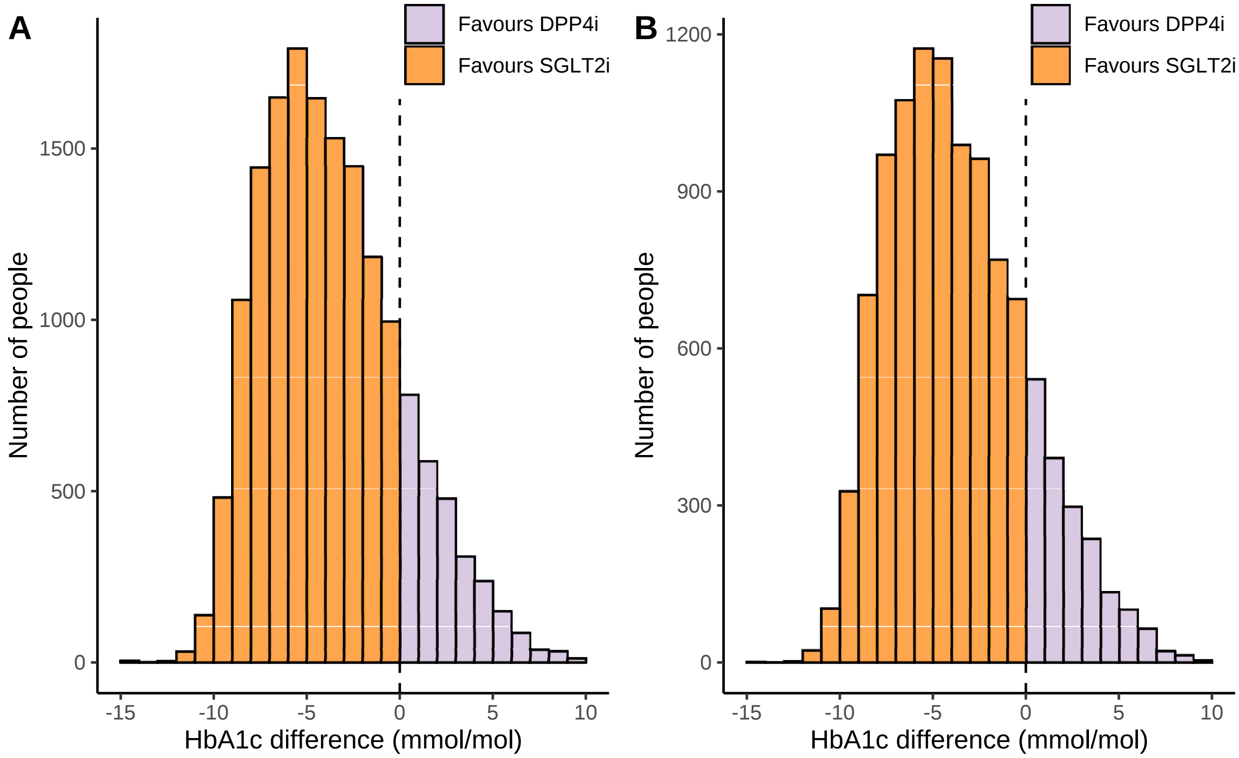

Supplementary Figure S7: Comparison of individualised treatment effects for SGLT2i and DPP4i treatments in the development (A) and validation (B) datasets. A negative value corresponds to a predicted glucose-lowering treatment benefit on SGLT2i and a positive value corresponds to a predicted glucose-lowering treatment benefit on DPP4i.

References

[1] Stefano Favaro and Stephen G. Walker. A generalized constructive definition for the Dirichlet process. *Statistics & Probability Letters*, 78(16), 2010.

[2] Jayaram Sethuraman. A constructive definition of Dirichlet priors. *Statistica Sinica*, 4(2):639–650, 1994.

[3] Chelsea Muth, Zita Oravecz, and Jonah Gabry. User-friendly Bayesian regression modeling: a tutorial with rstanarm and shinystan. *The Quantitative Methods for Psychology*, 14(2):99–119, 2018.

[4] Silvia Liverani, David I. Hastie, Lamiae Azizi, Michail Papathomas, and Sylvia Richardson. PReMiuM: An R package for profile regression mixture models using Dirichlet processes. *Journal of Statistical Software*, 64(7):1–30, 2015.

[5] John M. Dennis, Katherine G. Young, Andrew P. McGovern, Bilal A. Mateen, Sebastian J. Vollmer, Michael D. Simpson, William E. Henley, Rury R. Holman, Naveed Sattar, Ewan R. Pearson, Andrew T. Hattersley, Angus G. Jones, Beverley M. Shields, and on behalf of the MASTERMIND consortium. Development of a treatment selection algorithm for SGLT2 and DPP-4 inhibitor therapies in people with type 2 diabetes: a retrospective cohort study. *The Lancet Digital Health*, 4(12):e873–e883, 2022.

S4 MASTERMIND consortium

Prof Andrew Hattersley[1], Prof Ewan Pearson[2], Dr Angus Jones[1], Dr Beverley Shields[1], Dr John Dennis[1], Dr Lauren Rodgers[1], Prof William Henley[1], Prof Timothy McDonald[1], Prof Michael Weedon[1], Prof Nicky Britten[1], Catherine Angwin[1], Dr Naveed Sattar[3], Dr Robert Lindsay[3], Prof Christopher Jennison[4], Prof Mark Walker[5], Prof Kennedy Cruickshank[6], Dr Salim Janmohamed[7], Prof Christopher Hyde[1], Prof Rury Holman[8], Prof Andrew Farmer[8], Prof Alastair Gray[8], Prof Stephen Gough[8], Dr Olorunsola Agbaje[8], Dr Trevelyan McKinley[1], Dr Sebastian Vollmer[9], Dr Bilal Mateen[7], Prof William Hamilton[1]

[1]University of Exeter, Exeter, England

[2]University of Dundee, Dundee, Scotland

[3]University of Glasgow, Glasgow, Scotland

[4]University of Bath, Bath, England

[5]University of Newcastle, Newcastle upon Tyne, England

[6]Kings College London, London, England

[7]University College London, London, England

[8]University of Oxford, Oxford, England

[9]University of Kaiserslautern, Kaiserslautern, Germany
